# Pre-saccadic attention relies more on suppression than does covert attention

**DOI:** 10.1167/jov.23.1.1

**Published:** 2023-01-03

**Authors:** Julie Ouerfelli-Ethier, Isabella Comtois Bona, Romain Fournet, Laure Pisella, Aarlenne Z. Khan

**Affiliations:** 1 School of Optometry, University of Montreal, Montreal, Canada; 2 Lyon Neuroscience Research Center, Trajectoires team, University of Lyon I Claude-Bernard, Bron, France; 3 School of Optometry, University of Montreal, Montreal, Canada; 4 School of Optometry, University of Montreal, Montreal, Canada; 5 Lyon Neuroscience Research Center, Trajectoires team, University of Lyon I Claude-Bernard, Bron, France; 6 School of Optometry, University of Montreal, Montreal, Canada

**Keywords:** eye movement, target selection, inhibition, oculocentric map, priority map

## Abstract

During covert and pre-saccadic attentional shifts, it is unclear how facilitation and suppression processes interact for target selection. A recent countermanding task pointed to greater suppression at unattended locations during trials with saccades compared to trials without saccades (i.e., fixation and successful stop trials), whereas target facilitation did not differ. It is unknown whether this finding is restricted to countermanding paradigms that involve inhibitory processes. To test this, we adapted Gaspelin and colleagues (2015)’s attention capture task where, within the same block, one location was primed with frequent line discrimination trials, and all locations were occasionally probed using letters report trials. Participants also performed a baseline condition without priming. We tested 15 participants and examined how performance at non-primed locations was affected by covert versus pre-saccadic attention in blocks of four or six items, as well as by position from the primed location and timing from saccade onset. For both attention conditions, letter report at non-primed locations was worse compared to baseline, demonstrating suppression, and letter report at primed location was better, demonstrating facilitation. In saccades trials, letter report was better at primed locations and worse at non-primed locations compared to fixation trials. The timing of this additional pre-saccadic suppression differed from saccadic suppression. In both attention conditions, suppression was greater when primed and non-primed locations were within the same hemifield or in diagonal opposite quadrants. These results confirmed that attention preceding saccade execution suppressed non-primed locations to a larger extent than covert attention, with the same spatial quadrant effect.

## Introduction

Limited attentional resources imply that our system must prioritize in-depth visual processing for certain stimuli at the cost of others (Bisley & Goldberg, 2010; Fecteau & Munoz, 2006; Itti & Koch, 2001). The competition between attended and unattended locations can be observed with behavioral measures such as discrimination at these location or reaction times for movements toward these locations. Visual processing at the attended location, as well as movements to the attended location, are facilitated (i.e., better discrimination and shorter reaction times) (Brascamp, Pels, & Kristjánsson, 2011; Eriksen & Hoffman, 1973; Eriksen & Hoffman, 1974; Hoffman, 1975; Hoffman & Nelson, 1981; Kristjánsson & Ásgeirsson, 2019; Posner, 1980; Posner, Nissen, & Ogden, 1978; Posner & Cohen, 1984; Serences, Yantis, Culberson, & Awh, 2004; Sylvester, Jack, Corbetta, & Shulman, 2008). In contrast, suppression at unattended locations impedes discrimination and increases reaction times at these locations (Kastner & Pinsk, 2004; Pestilli & Carrasco, 2005; Posner & Cohen, 1984; Serences et al., 2004; Sylvester et al., 2008).

Facilitation and suppression processes occur in both covert (Gaspelin, Leonard, & Luck, 2015; Gaspelin & Luck, 2018; Sylvester et al., 2008) and pre-saccadic shifts of attention (Deubel & Schneider, 1996; Hoffman & Subramaniam, 1995; Kowler, Anderson, Dosher, & Blaser, 1995). However, whether these processes interact differently in covert and pre-saccadic shifts of attention remains unclear.

Different types of attentional competition may be characterized by different levels of facilitation and suppression or by different spatial and temporal modulation. The amount of remaining attentional resources across non-primed locations tends to vary with distance and hemifield effects. Unattended locations far from attended location are suppressed to a greater extent (Castiello & Umiltà, 1990; Laberge & Brown, 1986). Furthermore, at equidistance from the attended location, suppression at the unattended location within the same hemifield is enhanced compared to the hemifield opposite to the attended location (Khan, Munoz, Takahashi, Blohm, & McPeek, 2016). In addition, recent evidence has pointed to differences in temporal dynamics between covert and pre-saccadic shifts of attention (Li, Barbot, A., & Carrasco, 2016; Rolfs & Carrasco, 2012), as well as neuronal modulations (Baldassi & Verghese, 2005; Li, Pan, & Carrasco, 2021; Paltoglou & Neri, 2012). Note that although attentional suppression at non-primed locations in pre-saccadic conditions has been shown to be temporally modulated relative to saccade onset (e.g., Mikula, Jacob, Tran, Pisella, & Khan, 2018), it should not be confounded with the phenomenon of saccadic suppression, which has a shorter time scale around the saccade and concerns all space including saccadic goal location. Saccadic suppression is associated with poorer visual sensitivity when stimuli are presented less than 100 ms before saccade onset and during saccade, which aids in ignoring noisy visual information during the eye movement and helps us to perceive the world as stable (Diamond, Ross, & Morrone, 2000; Knöll, Binda, Concetta Morrone, & Bremmer, 2011; Latour, 1962; Volkmann, Riggs, White, & Moore, 1978; Zuber & Stark, 1966).

It has been shown that facilitation may either act similarly or differently for covert and pre-saccadic shifts of attention. On one hand, many studies have shown enhanced discrimination at saccade goal compared to unattended locations before saccades (Awh, Armstrong, & Moore, 2006; Deubel & Schneider, 1996; Findlay & Walker, 1999; Harrison, Mattingley, & Remington, 2013a; Hoffman & Subramaniam, 1995b; Kowler et al., 1995; McPeek & Keller, 2002). This facilitation effect has also been reported in covert shifts of attention (Kastner & Pinsk, 2004; Pestilli & Carrasco, 2005; Posner & Cohen, 1984; Serences et al., 2004; Sylvester et al., 2008). Furthermore, when compared across covert and pre-saccadic shifts of attention, discrimination at goal appears to be facilitated similarly for perception (Born, Ansorge, & Kerzel, 2013; Castet, Jeanjean, Montagnini, Laugier, & Masson, 2006; Deubel, 2008; Filali-Sadouk, Castet, Olivier, & Zenon, 2010; Khan, Blohm, Pisella, & Munoz, 2015; Rolfs & Carrasco, 2012). On the other hand, covert and pre-saccadic attention have different temporal dynamics for facilitation (Li et al., 2016; Rolfs & Carrasco, 2012). Covert and pre-saccadic attention can also be dissociated as the maintenance of attention does not require saccade programming (Belopolsky & Theeuwes, 2012) and covert attention may be oriented beyond the physical range of the eyes (Casteau & Smith, 2020; Hanning, Szinte, & Deubel, 2019; Smith, Schenk, & Rorden, 2012). This suggests that while behavioral components of facilitation may be similar across different attention shifts, some underlying neuronal processes may differ (Li, Hanning, & Carrasco , 2021).

Although perception may be facilitated similarly between covert and pre-saccadic shifts of attention, suppression may result in distinct perceptual patterns across different attentional shifts. A first support of this is that crowding is reduced at the saccade goal before the saccade occurs compared to when no saccade is performed (Harrison, Mattingley, et al., 2013a; Harrison, Mattingley, & Remington, 2013b; Harrison, Retell, Remington, & Mattingley, 2013), suggesting that pre-saccadic attention might be more efficient in suppressing target flankers. Furthermore, using a dual discrimination and countermanding task, Khan and colleagues (2015) demonstrated that saccade execution mechanisms suppress locations other than saccade goal. Greater suppression at uncued location was observed whenever a saccade was executed (in both go and failed stop trials) and was absent when no saccade was executed, both in covert attention (fixation) trials and during successfully stopped trials. This revealed a dissociation between selective attentional processes that may be common between covert and pre-saccadic conditions (Rizzolatti, Riggio, Dascola, & Umiltá, 1987; Rizzolatti & Craighero, 1998) and additional attentional processes associated with saccade execution, which may be mainly suppressive. However, it is unknown whether this suppression effect could be linked to the motor suppression processes required in this countermanding study (Khan et al., 2015).

Here we investigated how attentional suppression is modulated across covert and pre-saccadic conditions spatially and temporally in a paradigm without motor suppression. To do so, we adapted Gaspelin and colleagues (2015)’s attention capture task, where target selection was frequently primed with line discrimination trials, and attention at distractor locations was occasionally probed with letters identification trials within the same block. Using a version of this priming paradigm, we investigated whether and how the primed location is enhanced and how non-primed locations are suppressed with respect to the baseline condition and during saccades (overt) compared to fixation (covert) blocks. We further compared suppression patterns at non-primed locations as a function of relative position from the primed location and showed that it followed a similar spatial quadrant effect to that during covert attention shifts. Furthermore, we confirmed that this was attentional suppression rather than saccadic suppression by showing that suppression was not temporally linked to saccade onset.

## Materials and methods

### Participants

 We recruited 15 participants (*M* = 24 y, *SD* = 6 y, 11 females) from the community. Participants with neurological disorders, attentional deficits or taking medications that could affect attention (i.e., antidepressants or antiepileptic drugs, etc.) were excluded. All participants had normal or corrected-to-normal vision and gave informed written consent to participate in the experiment. They received financial compensation for their participation. Procedures received ethics approval from the Ethical Committee for Clinical Research at the University of Montreal.

We used G*power software (Faul, Erdfelder, Lang, & Buchner, 2007) to perform a power analysis of our statistical design with a repeated measures within factors *F*-test, with three groups and two measurements per group with an alpha of 0.05 and a beta of 0.8. The effect size 0.44 was calculated using a partial eta squared value of 0.164 from the analysis in the countermanding paper this article bases itself on (Khan et al., 2015). The total sample size provided was 15.

### Apparatus

Testing occurred at the University of Montreal (Montreal, Canada). Participants sat 57 cm away from a high-speed computer screen (20.5*11.5 inches, VIEWpixx 3D; VPixx Technologies, Montreal, Canada) in a dark room. Head movements were restricted with chin and forehead rests during the task. An eye-tracker recorded eye movements (EyeLink 1000 Plus; SR Research, Kanata, Canada; frequency: 1000 Hz). Participants entered their responses on a keyboard placed in front of them, which was illuminated by a lamp oriented such that it did not interfere with screen readability.

### Procedure

**Figure 1.**
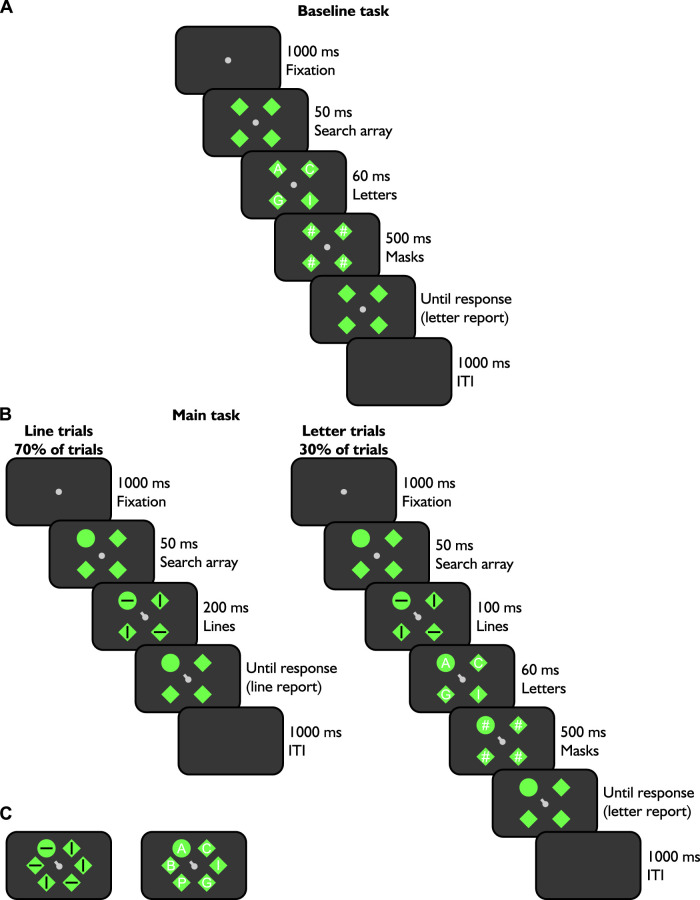
Experiment sequence of the baseline and main tasks. In A, we show the baseline task. The fixation dot remained alone on the screen, followed by placeholders. Then, letters flashed inside these shapes before being replaced by masks. Empty placeholders remained on the screen until participants’ response (letter report). During the main task (B), participants were presented with two types of trials: lines (left sequence, 70% of the time) and letters (right sequence, 30% of the time), randomly intermixed. Line trials started with a fixation dot screen before being replaced by placeholders: one green disk (i.e., cued location) and three green diamonds (i.e., non-cued locations). A cue originating from the fixation dot was always oriented toward the green disk. Next, differently oriented lines were presented within the placeholders. The cue, fixation dot, and placeholders were displayed until participants’ response (line orientation report). For letter trials, participants were first presented with fixation and placeholder screens. Lines were then flashed within the placeholders along with the cue pointing toward the primed location (i.e., green circle) and were then replaced with letters, which remained within the placeholders. Masks replaced the letters. The placeholder array remained on the screen until participants’ response. For these trials, participants had to report as many letters as possible, regardless of whether they appeared at primed (i.e., green circle) or non-primed (i.e., green diamonds) locations. In C is depicted the stimuli locations of six items blocks for both the line (left panel) and letter (right panel) trials.

Participants performed two tasks adapted from the attention capture task by Gaspelin and colleagues (2015): a baseline letter identification task where attention was not directed to one specific placeholder location (see Figure 1A), and the main task where attention was oriented to one specific placeholder location using a central cue (see Figure 1B). For both tasks, there were separate blocks of four and six placeholders (see Figure 1C). We tested participants on blocks of four and six items to vary task difficulty. All tasks were designed and implemented using MATLAB (The MathWorks, Inc., Natick, MA, USA) with Psychophysics toolbox (Brainard, 1997).

During the baseline task, participants maintained their fixation on a pale grey dot (dimensions: 0.5° by 0.5°) in the center of the screen against a dark grey background (luminance: 0.172 cd/m^2^). At the beginning of each trial, a fixation dot was presented alone for 1000 ms, followed by green diamonds as placeholders (dimensions: 2° by 2°; luminance: 61.053 cd/m^2^) displayed evenly around it at equidistance (4° from center) for 50 ms. Next, randomly selected (from all 26 letters) white capital letters (1° height) were presented centered in each shape for 60 ms. Hashtags of the same size served as masks and replaced the letters for 500 ms. Empty diamonds then remained on screen until participants’ response. Participants were instructed to identify as many letters as possible flashed within the placeholders. They could choose to report a minimum of one letter up to the number of displayed items (i.e., four or six) using a computer keyboard before pressing enter to go to the next trial. This screen was followed by an inter-trials interval of 1000 ms.

During the main task, participants were presented with two different types of trials randomly interleaved within a block: line (70% of trials, left panel in Figure 1B) and letter trials (30% trials, right panel in Figure 1B). The proportion of line and letter trials was determined to direct participants attention to a primed location (Gaspelin et al., 2015), such that we could probe letter report at both attended and unattended locations (i.e., non-primed locations). During line trials, participants discriminated the orientation of the line within the exogenously and endogenously cued disk target. With our paradigm, attention was allocated both exogenously because the primed location was a disk among diamonds and endogenously with central cueing. During letter trials, participants reported as many letters as possible regardless of the exogenously and endogenously cued disk target (30% trials, right panel on Figure 1B). Even if they were briefly presented with a screen containing lines within placeholders to prime one specific location, they only had to report letters during this type of trials. Priming of the green disk location is achieved through the lines search array in itself as line trials occurred frequently during blocks. Attention would thus be preferentially allocated to the cued location instead of the other locations.

At the beginning of each block, participants were instructed to either maintain fixation on the central dot (i.e., covert attention condition) or make a saccade to the cued position (i.e., pre-saccadic attention condition). They were also informed about the number of displayed items the block would contain: four or six items (see items configuration in Figures 1A–C, respectively). The beginning of the trial was identical to the baseline task: the color of the background, fixation screen, timings, but in this case, among the displayed empty placeholders there was one green disk (i.e., target/primed location) and green diamonds (i.e., distractor/non-primed locations). The location of the disk was additionally always indicated by a valid cue (pale grey line originating from the center of the fixation dot of a thickness of 0.3° and length of 0.5°). For line trials, the cue and lines of different random orientations (black lines of 0.3° thickness and 1° length) were presented in the placeholders at the same time for 200 ms. This was followed by an empty search array screen during which participants were instructed to indicate the orientation of the line contained in the cued disk on the arrows pad of the keyboard: up, for vertical lines, and down, for horizontal lines. For letters trials, the lines were displayed for 100 ms to prime participants’ attention to one location (i.e., green disk), and then were replaced by letters centered in each placeholder with the same size, timings, and instruction as for the baseline condition.

For the main task, blocks comprised 60 trials, and participants performed five blocks of the covert fixation task and six blocks of pre-saccadic saccade task for both four- and six-item blocks. We tested participants on one additional saccade block because we anticipated a greater number of saccade trials removed during data filtering. To prevent eye strain and fatigue in participants, testing occurred across two sessions of one hour each, and each testing session consisted of one baseline, five fixation, and six saccade blocks. Half of the participants (*n* = 7) first performed four-item blocks whereas the other half (*n* = 7) started with six-item blocks. The order of the fixation and saccade blocks were randomized in the first session and tested in reverse order in the second session for these participants. For the fifteenth participant, the order of their blocks for the first session was randomized across both fixation and saccade condition and number of item trials. They were then tested in the reverse order in the second session. In addition to the 11 blocks participants performed per session, they did the baseline task, which was the last block in the first session and the first block of the second session for all participants; the item condition was determined by whether they started with the four- or six-item blocks and was selected randomly for the fifteenth participant.

### Preliminary analyses

For the baseline task, we recorded a total of 900 trials. Saccade timing and position were automatically calculated offline using a saccade detection algorithm with a velocity criterion of 15°/s where time points such as the beginning of the trial, letter onset, and line onset were also indicated. We could thus visually examine each trial to remove trials during which eye signal was lost during stimuli presentation as a result of blinks or the tracker losing the eye position (five trials, 0.56% of total number of trials). We then removed all trials with saccades made 500 ms after letter presentation (in which cases, attention could have been allocated covertly to any location before a saccade was planned and executed to the primed location). This represented 103 trials, or 11.44% of the total number of trials. There remained 792 trials for further analyses.

For the main task, we recorded a total of 19,800 trials. As for the baseline task, we first conducted a visual inspection of each trial to remove those with loss of eye signal during item presentation (223 trials, 1.13% of total number of trials). For the pre-saccadic condition, we calculated saccade reaction time as saccade onset relative to line onset. We then removed trials with saccade reaction times below 100 ms to exclude anticipatory and express saccades (Fischer & Ramsperger, 1984, 1986; Fischer & Weber, 1997; Mayfrank, Mobashery, Kimmig, & Fischer, 1986; Weber, Aiple, Fischer, & Latanov, 1992). We also excluded trials with saccade reaction times over 500 ms (552 trials, 2.79% of total number of trials). We removed these trials because we considered that with longer saccade reaction times, participants might be waiting to make the saccade and instead allocated their attention covertly at a location shortly after letter presentation before planning and executing a saccade to the primed location. We also filtered out trials with saccades smaller than 2° and larger than 6° amplitudes and those made outside of 20 angular degrees from the primed location. For the fixation condition, we removed trials where saccades were made with an amplitude larger than 2° or with saccades made within 500 ms of lines or letters onset. This represented 1524 trials, and there remained 17,501 trials (88.39% of total number of trials).

With the remaining trials, we analyzed task performance solely for letters trials, where we calculated the mean percentage of correct letter reports per condition (i.e., baseline, fixation, and saccade) and number of items (i.e., four and six). We only analyzed trials where the saccade began after the line/letter disappeared. To do so, we divided the number of correctly identified letters over total number of displayed letters (four or six) for baseline, saccade, and fixation conditions and for four or six items separately. We further calculated chance levels per participant. Chance is the number of choices out of 26 possibilities (26 letters). If there were 26 choices possible, then chance performance would be 100%. For example, if a participant reported two letters in one trial, then accuracy should be compared to a chance level of 2/26. If in another trial the participant reported four letters, then accuracy should be compared to a chance level of 4/26. Therefore chance level depended on the mean number of responses the participant made rather than the number of items presented in the block. Each participant could respond with one guess up to the number of items in the block (four or six maximum), and letters within one trial could not be repeated. We calculated the mean number of responses for each participant for each condition and item number and divided this number by 26 (i.e., total number of potential responses). We used chi-square tests to compare performance at non-primed locations for the main and baseline tasks to chance. This analysis allowed us to confirm we were indeed measuring attentional suppression at these locations. If performance at non-primed locations were not different from chance levels, we could not distinguish between performance being the result from suppression or an absence of attention deployed at these locations.

We also compared baseline to overall performance at both primed and non-primed locations for fixation and pre-saccadic trials with paired sample *t*-tests. Of note, while performance at non-primed locations appear to be lower during six item blocks compared to four item blocks, these performances reflected similar mean number of letter report for both fixation (*M*_four items_ = 1.26 letters; *M*_six items_ = 1.16 letters) and pre-saccadic conditions (*M*_four items_ = 1.02 letters; *M*_six items_ = 1 letter). Letter report at baseline also appeared comparable between four (*M* = 1.83 letters) and six item blocks (*M* = 1.94 letters). The difficulty of the task was rather ascertained with the number of correct answers relative to the total number of items.

With repeated measures analyses of variance (ANOVAs), we next examined overall performance across primed and non-primed locations, task difficulty (i.e., four- and six-items blocks) and our two attention conditions (i.e., fixation and pre-saccadic). Furthermore, we tested how relative position from primed location affected performance at non-primed locations. To do so, we collapsed performance at non-primed locations according to the quadrant where they were in relative to the primed location: orthogonal position within the same hemifield, orthogonal position in the opposite hemifield, and diagonally across. These analyses tested performance at non-primed locations across task difficulty (four vs. six items), attention condition (pre-saccadic vs. fixation) and quadrant as main effects.

Using only the saccade trials, we conducted temporal analysis where we subtracted saccade onset from letter onset to compare letter discrimination performance when letter onset occurred shortly before (i.e., bin < 100 ms) or long before saccade onset (i.e., bin > 100 ms) at primed and non-primed locations. In this analysis, we included trials in which saccade onset occurred before line/letter offset (13.02% of trials). We compared performance as a function of saccade onset with paired sample *t*-tests. This temporal analysis was designed specifically to explore whether performance at non-primed location could be explained by saccadic suppression. In this case, performance would be expected to be lower when letter onset occurred shortly before saccade onset (within peri-saccadic window) as opposed to long before saccade onset.

For all our analyses, any significant main effect was followed up by Holm-Bonferroni corrected post-hoc comparisons adjusted familywise. ANOVA degrees of freedom were Greenhouse-Geiser corrected if Mauchly's test of sphericity was violated. We conducted all statistical analyses on JASP 0.16.3.0 (JASP Team, 2022).

## Results

We compared performance to chance levels to ensure that attention was deployed to primed and non-primed locations (be it minimal, allowing evidence for suppression rather than an absence of attention). We also compared overall performance for baseline condition and the two main tasks at both primed and non-primed locations. This allowed us to explore whether participants performed the task by orienting attention to the primed location, and whether performance was facilitated or suppressed compared to baseline at non-primed locations. We investigated performance at non-primed locations relative to the quadrant that contained the primed location. Finally, we conducted a temporal analysis to investigate the possibility that the pattern of suppression we observed could be explained by saccadic suppression.

### Chance levels

With chi-square goodness of fit tests, we examined whether participants’ proportion of percentage correct and incorrect was equal or not between chance levels and non-primed locations. We compared these proportions for both fixation and saccade, and four- and six-item blocks separately. Our results showed that proportions of performance were significantly different for each attention condition and for both four- (*χ^2^fixation* = 18.714, *p* = 0.00002; *χ^2^saccade* = 13.781, *p* = 0.0002) and six-item blocks (*χ^2^fixation* = 122.464, *p* < 0.00001; *χ^2^saccade* = 4.735, *p* = 0.030) compared to chance levels. In other terms, participants’ performance at non-primed locations differed from chance levels during our main task.

### Baseline task

**Figure 2.**
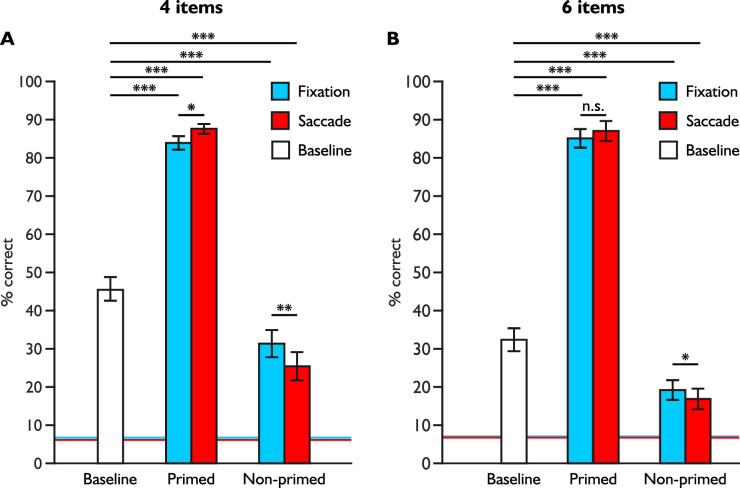
Overall performance for the baseline and main tasks. We present overall performances for four (A) and six (B) items blocks at primed and non-primed locations for letter identification, baseline and chance levels. The fixation condition is illustrated in blue, the saccade condition in red, and the baseline condition in white. Statistical differences resulted from Holm-Bonferroni corrected post hoc paired sample *t*-tests. Mean chance levels appear as blue and red lines for respectively fixation and saccade blocks. Error bars correspond to standard errors of the mean. n.s., not significant, ***, *p* < 0.001, Holm-Bonferroni corrected.

We compared performance in the main task to the baseline task; if attention shifts facilitate letter identification at the cued primed location, overall performance at this location should be higher than overall baseline performance. Similarly, if attention shifts to primed location suppress non-primed locations, overall performance at these locations should be lower than overall baseline performance. We illustrated overall baseline performance in white whereas the fixation condition is depicted in blue, and the pre-saccadic condition in red in Figure 2.

Our hypotheses were confirmed with Holm-Bonferroni corrected paired sample *t*-tests: baseline performance was significantly lower compared to performance at primed location for both fixation and pre-saccadic conditions, and for both four- (*t*[14] = −14.991, *p* < 0.001, *d* = −3.871; *t*(14) = −16.685, *p* < 0.001, *d* = −4.308), and six-item blocks (*t*[14] = −26.256, *p* < .001, *d* = −6.779; *t*(14) = −19.043, *p* < .001, *d* = −4.917). Inversely, baseline performance was significantly higher compared to performance at non-primed locations, and this was the case for both fixation and pre-saccadic conditions, and for both four- (*t*[14] = −4.433, *p* < 0.001, *d* = 1.145; *t*[14] = 6.785, *p* < 0.001, *d* = 1.752) and six-item blocks (*t*[14] = 6.732, *p* < 0.001, *d* = 1.738; *t*(14) = 8.493, *p* < 0.001, *d* = 2.193). In conclusion, our results suggest that performance at primed location was facilitated compared to baseline, and that performance at non-primed locations was suppressed compared to baseline.

### Overall performance

We first confirmed that participants were correctly allocating attention to the primed location during line trials: discrimination performance was almost perfect during both fixation and saccade conditions and four- (*M* = 99.07%, *SD* = 0.79%; *M* = 99.19%, *SD* = 1.12%) and six-item blocks (*M* = 98.58%, *SD* = 1.19%; *M* = 98.26%, *SD* = 2.08%). Subsequent analyses concerned only letter trials. With a three-way repeated measures ANOVA, we compared overall performance across task difficulty (i.e., four and six items), attention condition (i.e., fixation and pre-saccadic) and location (i.e., primed and non-primed) (see Figure 2).

We found main effects for task difficulty (*F*[1, 14] = 10.556, *p* = 0.006, η^2^_p_ = .430) and location (*F*[1, 14] = 466.259, *p* < 0.001, η^2^_p_ = .971) but no main effect for condition (*F*[1, 14] = 0.888, *p* = 0.362, η^2^_p_ = .060) (see Figure 2A). Interactions effects between task difficulty and location (*F*[1, 14] = 22.066, *p* < 0.001, η^2^_p_ = .612) and between attention condition and location (*F*[1, 14] = 13.511, *p* = 0.002, η^2^_p_ = .491) were also significant. In other words, performance at primed and non-primed locations varied according to attention condition and according to task difficulty. However, the interaction between task difficulty and attention condition along with the three-way interaction were not significant (*F*[1, 14] = 0.154, *p* = 0.70, η^2^_p_ = 0.011; *F*[1, 14] = 3.263, *p* = 0.092, η^2^_p_ = 0.189).

Post hoc comparisons with Holm-Bonferroni corrections revealed that performance at the primed location was significantly higher during the saccade condition compared to the fixation condition (*t*[14] = −2.130, *p* = 0.042, *d* = −0.258). In contrast, we found significantly lower performance at non-primed locations during saccades compared to during fixation (*t*[14] = 3.378, *p* = 0.004, *d* = 0.409). Regardless of attention condition, performance at primed locations was significantly higher than non-primed locations (*p* < .001, *d* > 5.688). Performance during six-item blocks was significantly lower compared to four-item blocks at non-primed location (*t*[14] = 5.375, *p* < 0.001, *d* = 1.006). Performance at non-primed locations was also significantly lower than at primed location regardless of task difficulty (*p* < .001, *d* > 5.527). In comparison, performance at primed locations did not differ between four- and six-item blocks (*t*[14] = −0.090, *p* = 0.929, *d* = −0.017).

Overall, we found lower performance with increased tasks difficulty particularly for non-primed locations comparisons and higher performance at primed location compared to non-primed locations. Furthermore, performance at non-primed locations was more suppressed before saccades than during fixation.

### Quadrant performance

**Figure 3.**
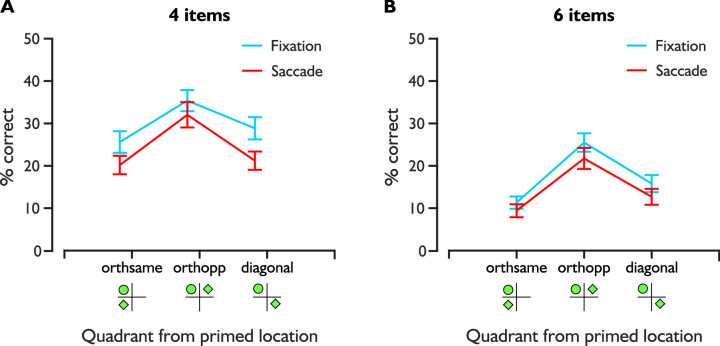
Non-primed locations relative to primed location quadrant. The fixation condition is represented in blue and the saccade condition, in red. We show in A and B the mean percentage correct for letter report in non-primed locations (illustrated as green diamonds) relative to the quadrant containing the prime (illustrated as a green disk): orthogonal in the same hemifield, orthogonal in the opposite hemifield or diagonal. Performance is displayed for four item blocks in A and for six item blocks in B. In both graphs, errors bars are standard errors of the mean.

We investigated how performance at non-primed locations was affected by the presence of the primed location in the orthogonal quadrant within the same or opposite hemifield or in the diagonal across quadrant. For the orthogonal quadrant in the opposite hemifield during six item blocks, we distinguished between items which were opposed to each other on the horizontal axis (i.e., 180° away) and items at other non-primed locations (i.e., 60° away) because of the horizontal attentional bias, which may affect results (Mackeben, 1999). For this reason, we conducted our analyses without the case of primed/non-primed on the horizontal line in the orthogonally opposite quadrant. To account for this bias and to match as closely as possible the stimuli layout of the two number of items blocks, we also excluded trials where the primed or non-primed location fell on one of the two horizontal meridian positions during six-item blocks. We illustrated our findings in Figure 3. Following the same color code as previously stated.

We performed a three-way repeated measures ANOVA with task difficulty, covert (fixation) and pre-saccadic attention conditions, and quadrant as factors for performance at non-primed locations. In terms of quadrant, these non-primed locations were classified as orthogonal to the primed location within the same (orthogonal same) or opposite hemifield (orthogonal opposite) or diagonal relative to the primed location (diagonal).

We found main effects of task difficulty (*F*[1, 14] = 32.388, *p* < 0.001, η^2^_p_ = .698), attention condition (*F*[1, 14] = 11.546, *p* < 0.004, η^2^_p_ = .452), and quadrant (*F*[2, 28] = 13.343, *p* < 0.001, η^2^_p_ = .488). There was a trend toward significance for the interaction between task difficulty and attention condition (*F*[1, 14] = 4.125, *p* = 0.062, η^2^_p_ = .288). The remaining interactions also did not reach significance (*p* > .307, η^2^_p_ < .081). Holm-Bonferroni corrected post hoc comparisons revealed significantly higher performance in the orthogonal opposite quadrant compared to performance in both the orthogonal same quadrant (*t*[14] = −4.967, *p* < 0.001, *d* = −0.839) and the diagonal quadrant (*t*[14] = 3.714, *p* = 0.002, *d* = 0.627). Performance in the orthogonal same and diagonal quadrants was not significantly different (*t*[14] = −1.252, *p* = 0.221, *d* = −0.211).

Overall, the significant main effect of attention condition confirms our previous analysis where performance at non-primed locations was more suppressed before saccades compared to during fixation. In addition, we showed that this pattern of suppression was similar across attention condition and task difficulty.

### Performance as a function of saccade onset

We explored how performance was affected by letter onset relative to saccade onset separately for four- and six-item blocks. This analysis aimed to probe whether there was a temporal fluctuation in performance that would be consistent with the timing of saccadic suppression. Saccadic suppression is associated with poorer visual sensitivity when stimuli are presented 100 ms or less before saccade onset (e.g., Diamond et al., 2000).

For this temporal analysis, we calculated the time of letter onset from saccade onset (Diamond et al., 2000). We then divided these timings in two bins: when letter onset occurred less than and more than 100 ms before saccade onset. If lower discrimination performance was due to saccadic suppression, we would expect that it would be poorer only when time from saccade onset was less than 100 ms. We removed one participant from the analysis, who had fewer than five trials per bin for <100 ms for both four- and six-item blocks. Mean and *SD* performance and trial numbers per bin per participant are available in Supplementary Table S1. For four-item blocks, Holm-Bonferroni corrected paired sample *t*-tests performed showed no significant differences between the two bins at primed (*t*[13] = −2.387, *p* = 0.132, *d* = −0.638) and non-primed locations (*t*[13] = 1.655, *p* = 0.122, *d* = 0.442). We found the same pattern for six-item blocks (*t*[13] = −0.680, *p* = 0.508, *d* = −0.182; *t*(13) = −0.068, *p* = 0.947, *d* = −0.018). In sum, there was thus no evidence of letter discrimination being poorer shortly before saccade onset.

## Discussion

We investigated facilitation and suppression comparing covert and pre-saccadic attention conditions, the number of items and the relative quadrants of non-primed locations. In both attention conditions, we found facilitation at the primed location and suppression at non-primed locations compared to a baseline task without priming. Furthermore, we observed that suppression at non-primed locations was greater with increasing number of items but followed a similar spatial quadrant pattern across attention conditions and task difficulty. This similar spatial quadrant pattern may reflect common suppression processes underlying attention allocation for both covert and pre-saccadic conditions. However, we observed that non-primed locations were overall suppressed to a larger extent before saccades compared to fixation blocks replicating past results from our group in the context of a countermanding task (Khan et al., 2015). Unlike Khan et colleagues (2015)’s study, we observed enhanced facilitation at primed location across task difficulty in the pre-saccadic condition compared to the covert attention condition.

On one hand, our results of enhanced performance at the primed location along with decreased performance at non-primed locations for both covert and pre-saccadic conditions compared to baseline condition is consistent with findings from previous studies. For example, studies have shown enhancement at attended location for fixation and saccade trials (Born et al., 2013; Castet et al., 2006; Deubel, 2008; Filali-Sadouk et al., 2010; Khan et al., 2015; Li, Pan, et al., 2021; Rolfs & Carrasco, 2012). Suppression of unattended locations has been demonstrated concomitant to attended location enhancement before saccades (Deubel & Schneider, 1996; Findlay & Walker, 1999; Khan et al., 2015; Khan et al., 2016; Kowler et al., 1995; Schlag et al., 1998; Schlag-Rey et al., 1992) and during fixation (Kastner & Pinsk, 2004; Khan et al., 2015; Khan et al., 2016; Pestilli & Carrasco, 2005; Posner & Cohen, 1984; Serences et al., 2004; Sylvester et al., 2008). Furthermore, we observed in the present results a specific spatial suppression pattern of significantly lower letter report in the orthogonal same quadrant compared to the orthogonal opposite quadrant with respect to primed location during both covert and pre-saccadic attentional conditions. This spatial pattern of suppression suggests that letter report varied as a function of both hemifield and distance in both instances. For non-primed locations at the same distance from the primed location, suppression was higher when both were within the same hemifield, compared to when they were in opposite hemifields. Similar findings have been observed in previous work (Deubel & Schneider, 1996; Khan et al., 2016; Kowler et al., 1995; Rizzolatti et al., 1987) and could be a result of additional competition from irrelevant locations for selection within the same hemifield (McSorley, Haggard, & Walker, 2004). For non-primed locations within the opposite hemifield to the primed location, suppression varied such that the most remote location to the attended location (i.e., diagonally opposite quadrant) was more suppressed than closer locations (i.e., orthogonal opposite quadrant). Previous studies similarly have shown lower discrimination at far or diagonally opposite locations to the attended location for both covert and pre-saccadic shifts of attention (Khan et al., 2015) and reduced visual sensitivity at farther locations relative to the attended location (Astrand, Wardak, & ben Hamed, 2020). Taken together, these findings suggest common processes of enhancement and suppression.

This similar pattern of performance likely reflects shared underlying neuronal mechanisms for pre-saccadic and covert attention shifts. Several brain areas, such as the superior colliculus (SC), the frontal eye field (FEF), and the posterior parietal cortex (PPC) have been implicated in processes involving the orienting of attention, as well as eye movements (Bogadhi, Bollimunta, Leopold, & Krauzlis, 2018; Bollimunta, Bogadhi, & Krauzlis, 2018; Goldberg et al., 2006; McPeek & Keller, 2002; Thompson & Bichot, 2005; Wang et al., 2022). These brain areas contain oculo-centric maps of visual space, which are considered to embody priority maps for both covert and pre-saccadic attention (Bayguinov et al., 2015; Bisley & Goldberg, 2010; Bisley & Mirpour, 2019; Fecteau & Munoz, 2006; Mirpour et al., 2009; Ptak, 2012; Thompson & Bichot, 2005). In priority maps, neurons representing different locations of the visual field containing relevant and irrelevant stimuli compete for selection based on exogenous and endogenous relevance (Dhawan et al., 2013; Fecteau & Munoz, 2006; Findlay & Walker, 1999; Geng, 2014; Itti & Koch, 2000, 2001). This competition in priority maps translates into concurrent neuronal enhancement for representation of the relevant object's location, and suppression, or spatial-based inhibition, of the representation of the irrelevant objects’ locations (Awh et al., 2006; Kastner et al., 1998; McPeek & Keller, 2002; McSorley et al., 2006; Reynolds et al., 1999; Serences et al., 2004). Because they contain oculocentric priority maps, the FEF, PPC and SC have properties that could subtend common enhancement and suppression processes in both covert and pre-saccadic attention tasks.

Neuronal properties in FEF are consistent with priority maps (Bisley & Mirpour, 2019) implicated in both covert and pre-saccadic attention (Bogadhi et al., 2018; Bollimunta et al., 2018; Moore & Fallah, 2001; Moore & Fallah, 2004; Murthy, Thompson, & Schall, 2001; Schall, 2002; Schall, Hanes, Thompson, & King, 1995; Sommer & Wurtz, 2000; Thompson, Biscoe, & Sato, 2005). In FEF, potential target related-activity is enhanced and maintained to facilitate target selection during gaze and attention shifts (Armstrong, Chang, & Moore, 2009; Bichot & Schall, 1999; Moore & Fallah, 2001; Schlag-Rey et al., 1992; Sommer & Wurtz, 2000) while distractor-related activity is concurrently suppressed (Schlag-Rey, Schlag, & Dassonville, 1992; Sommer & Wurtz, 2000; Suzuki & Gottlieb, 2013). Both microstimulation and transcranial magnetic stimulation applied to the FEF increase distractor interference during visual search as well as bias saccades away from distractors (McPeek, 2006; Walker, Techawachirakul, & Haggard, 2009; Wardak, Ibos, Duhamel, & Olivier, 2006). Saccades curved toward distractors are accompanied by increased activity in FEF whereas saccades curved away from distractors are accompanied by decreased activity, which suggests that suppression processes can modulate saccade metrics and target selection (McPeek, 2006). FEF activity is also predictive of successful distractor inhibition (Sakai, Rowe, & Passingham, 2002). Similarly, FEF inactivation leads to distractor interference during visual search without eye movement, which results in poorer performance (Wardak et al., 2006). Evidence from transcranial magnetic stimulation applied to the FEF also points to a role in the inhibition of irrelevant stimuli (Ro, Farnè, A., & Chang, 2003; Smith, Jackson, & Rorden, 2005). FEF thus shows similar enhancement and suppression processes during covert and pre-saccadic shifts of attention which could contribute to explain our results.

Like the FEF, the PPC is a region involved in both covert and pre-saccadic spatial attention (Blangero, Khan, Rode, Rossetti, & Pisella, 2011; Bogadhi et al., 2018; Gillebert, Mantini, Thijs, Sunaert, Dupont, & Vandenberghe, 2011; Khan, Blangero, Rossetti, Salemme, Luauté, Deubel, Schneider, Laverdure, Rode, Boisson, & Pisella, 2009; Striemer, Blangero, Rossetti, Boisson, Rode, Vighetto, Pisella, & Danckert, 2007; Striemer, Locklin, Blangero, Rossetti, Pisella, & Danckert, 2009) and priority maps are hypothesized to be implemented there (Bisley & Goldberg, 2010). Although the PPC has been primarily linked to perceptual enhancements during saccade and non-saccade conditions (Reynolds & Chelazzi, 2004), inhibitory processes across hemifields have also been observed (Belmonte, 1998; Belmonte & Yurgelun-Todd, 2003; Müller, Teder-Sälejärvi, & Hillyard, 1998), for example, through the filtering of irrelevant locations during object selection (Friedman-Hill, Robertson, Desimone, & Ungerleider, 2003). Moreover, patients with lesions to the PPC show more inhibitory deficits in their affected hemifield (Blangero et al., 2011; Friedman-Hill et al., 2003; Ouerfelli-Ethier, Salemme, Fournet, Urquizar, Pisella, Khan, & Khan, 2021) in line with theories of interhemispheric competition for attentional control (Śmigasiewicz, Weinrich, Reinhardt, & Verleger, 2014; Szczepanski, Konen, & Kastner, 2010; Szczepanski & Kastner, 2013). The suppression pattern we found may thus rely on PPC activity modulation during both covert and pre-saccadic shifts of attention.

Alongside FEF and PPC, the SC is involved in both covert and pre-saccadic shifts of attention (Bogadhi et al., 2018; Bollimunta et al., 2018; Ignashchenkova, Dicke, Haarmeier, & Thier, 2003; Krauzlis, Lovejoy, & Zénon, 2013; McPeek & Keller, 2004; Wang, Herman, & Krauzlis, 2022) where its overall role could be target selection (Krauzlis, Liston, & Carello, 2004). In its intermediate and deep layers, target-related activity is enhanced, regardless of whether a saccade is executed or not (McPeek & Keller, 2002; Wang et al., 2022). Accordingly, pharmacological inactivation affects attention through target detection (Bollimunta et al., 2018) and biases saccades toward distractors (McPeek & Keller, 2004; Nummela & Krauzlis, 2010). It was hypothesized that competing signals from target and distractors within the SC biases responses (McPeek & Keller, 2004) are in line with the implementation of priority maps in this structure (Fecteau & Munoz, 2006). Further support for the SC as a good candidate to explain our results comes from the form of competition related to visual and motor signals based on known short-distance excitatory and long-distance inhibitory neuronal connectivity within the SC (Dorris, Olivier, & Munoz, 2007; Khan, Munoz, Takahashi, Blohm, & McPeek, 2016; Marino, Trappenberg, Dorris, & Munoz, 2012; Munoz & Fecteau, 2002). Patterns of distractor-target interactions dependent on quadrants could naturally arise from this known inherent network properties of the SC, simulated by the 2D dynamic field model (Marino et al., 2012). This model incorporates the short-distance excitatory and long-distance inhibitory connections in two dimensions and could result in a certain pattern of competition depending on the spatial relationship between the distractor and the saccade target both as a function of distance and direction. In their study, Khan and colleagues (2016) accordingly showed that attentional modulation at the cued location (due to a cue and a target appearing at varying stimulus onset asynchrony (SOA)) was accompanied by saccade reaction times varying as a function of quadrant. Our findings highlight a suppression pattern depending on quadrant, which is consistent with an interpretation of competitive interaction in terms of lateral connectivity in the SC for both covert and pre-saccadic attention.

On the other hand, even though the spatial pattern of attentional modulation across the visual field was similar for covert and pre-saccadic shifts of attention, we observed different levels of attentional enhancement and suppression between the two attentional conditions, i.e. enhanced facilitation at primed location and enhanced suppression at non-primed locations before saccades compared to during fixation. In Khan and colleagues (2015)’s study, enhanced facilitation at the goal location before saccades compared to fixation was not observed, likely because of the inhibitory nature of their countermanding task. Their task could have led to an overall suppression effect that resulted in equally impeded performance at primed location and further suppression at non-primed locations or to a specific suppression effect at the primed location. Nevertheless, suppression, both in the present study and in Khan et colleagues (2015)’s study, was consistently greater in pre-saccadic condition, both overall and independently of quadrant and task difficulty. These findings imply that saccade planning leads to additional perceptual suppression at non-primed locations. One possible explanation could be saccadic suppression, where shortly before and during saccades visual sensitivity is reduced (Diamond et al., 2000; Knöll et al., 2011; Latour, 1962; Volkmann et al., 1978; Zuber & Stark, 1966). In accordance, there is evidence for a close functional and temporal relationship between oculomotor and visual sensory areas in the primate brain, providing an anatomical basis for how saccade planning processes in FEF can influence the activity of visual neurons in V4 involved in perception at the saccadic goal location (Gregoriou, Gotts, & Desimone, 2012; Moore & Armstrong, 2003; Wurtz & Mohler, 1976). However, we did not observe an additional attentional suppression in the 100 ms preceding saccade onset, as would then be expected. If the additional inhibitory effect we observed at non-primed locations was guided by saccadic perceptual suppression in the pathway from FEF to visual cortex, we would have obtained lower performance for saccade onset that occurred shortly after letter onset compared to much later after.

Another possible explanation for the additional suppression in pre-saccadic attention observed in this article is due to saccade-specific suppression in the pathway from FEF to SC. It has been shown that distinct neuronal populations in FEF contribute to attentional shifts and saccade execution (Brown, Hanes, Schall, & Stuphorn, 2008; Juan, Shorter-Jacobi, & Schall, 2004; Ray, Pouget, & Schall, 2009; Thompson, Bichot, & Schall, 1997; Thompson & Bichot, 2005; Wardak, Olivier, & Duhamel, 2011). Only saccade-related neuronal populations in the FEF may therefore play a key role in the suppression of locations competing with saccade goal across the SC maps (Mysore & Knudsen, 2011; Mysore & Knudsen, 2013). The observed additional inhibitory mechanism during saccades may therefore be a result of extrinsic GABAergic input from oculomotor areas such as substantia nigra pars reticulata, zona incerta, and mesencephalic reticular formation to the SC (Appell & Behan, 1990; Lai, Brandt, Luksch, & Wessel, 2011; Mysore, Asadollahi, & Knudsen, 2010; Wang, Major, & Karten, 2004) linked specifically to saccade planning (Chometton, Charrière, Bayer, Houdayer, Franchi, Poncet, Fellmann, & Risold, 2017; Cromer & Waitzman, 2006; Hikosaka & Wurtz, 1983; Mitrofanis, 2005). For example, projections from the substantia nigra pars reticulata of the basal ganglia (Hikosaka & Wurtz, 1985) provide global inhibition throughout the SC map, through a gating mechanism specific to saccades (Keller, 1974; Keller, 1977; Khan, Blohm, McPeek, & Lefèvre , 2009; Luschei & Fuchs, 1972). These saccade specific inhibitory mechanisms may therefore account for the additional inhibition we observed during the condition of pre-saccadic attention.

## Conclusion

To conclude, we found similar patterns of enhancement at the primed location and suppression at non-primed locations for covert and pre-saccadic attention. This is consistent with past neurophysiological findings on oculocentric priority maps in the SC, PPC and FEF, where covert and pre-saccadic attention share common neuronal mechanisms. Suppression processes appeared to vary similarly as a function of quadrant across covert and pre-saccadic attention conditions, which is consistent with the 2D dynamic field model based on known inherent network properties of the SC. Our results also revealed an additional level of enhancement and suppression in pre-saccadic attention which could be explained by distinct neuronal populations within the cortex underlying saccades and visual attention, and specific subcortical circuits inhibiting the SC map in pre-saccadic attention. Taken together, it is likely that both cortical and subcortical circuitry contribute, independently or competitively, to shared and different mechanisms involved in covert and pre-saccadic attention.

## Supplementary Material

Supplement 1

## References

[bib1] Appell, P. P., & Behan, M. (1990). Sources of subcortical GABAergic projections to the superior colliculus in the cat. *Journal of Comparative Neurology,* 302(1), 143–158, 10.1002/CNE.903020111. 2086611

[bib2] Armstrong, K. M., Chang, M. H., & Moore, T. (2009). Selection and maintenance of spatial information by frontal eye field neurons. *Journal of Neuroscience,* 29(50), 15621–15629, 10.1523/JNEUROSCI.4465-09.2009. 20016076 PMC3351279

[bib3] Astrand, E., Wardak, C., & ben Hamed, S. (2020). Neuronal population correlates of target selection and distractor filtering. *NeuroImage,* 209, 116517, 10.1016/J.NEUROIMAGE.2020.116517. 31923605

[bib4] Awh, E., Armstrong, K. M., & Moore, T. (2006). Visual and oculomotor selection: links, causes and implications for spatial attention. *Trends in Cognitive Sciences,* 10(3), 124–130, 10.1016/J.TICS.2006.01.001. 16469523

[bib5] Baldassi, S., & Verghese, P. (2005). Attention to locations and features: Different top-down modulation of detector weights. *Journal of Vision,* 5(6), 7–7, 10.1167/5.6.7. 16097868

[bib6] Bayguinov, P. O., Ghitani, N., Jackson, M. B., & Basso, M. A. (2015). A hard-wired priority map in the superior colliculus shaped by asymmetric inhibitory circuitry. *Journal of Neurophysiology,* 114(1), 662–676, 10.1152/JN.00144.2015/ASSET/IMAGES/LARGE/Z9K0121531620007.JPEG. 25995346 PMC4512250

[bib7] Belmonte, M. (1998). Shifts of visual spatial attention modulate a steady-state visual evoked potential. *Cognitive Brain Research,* 6(4), 295–307, 10.1016/S0926-6410(98)00007-X. 9593953

[bib8] Belmonte, M. K., & Yurgelun-Todd, D. A. (2003). Anatomic dissociation of selective and suppressive processes in visual attention. *NeuroImage,* 19(1), 180–189, 10.1016/S1053-8119(03)00033-8. 12781737

[bib9] Belopolsky, A. v., & Theeuwes, J. (2012). Updating the premotor theory: The allocation of attention is not always accompanied by saccade preparation. *Journal of Experimental Psychology: Human Perception and Performance,* 38(4), 902–914, 10.1037/A0028662. 22686694

[bib10] Bichot, N. P., & Schall, J. D. (1999). Effects of similarity and history on neural mechanisms of visual selection. *Nature Neuroscience,* 2(6), 549–554, 10.1038/9205. 10448220

[bib11] Bisley, J. W., & Goldberg, M. E. (2010). Attention, Intention, and Priority in the Parietal Lobe. *Annual Review of Neuroscience,* 33(1), 1–21, 10.1146/annurev-neuro-060909-152823. PMC3683564 20192813

[bib12] Bisley, J. W., & Mirpour, K. (2019). The neural instantiation of a priority map. *Current Opinion in Psychology,* 29, 108–112, 10.1016/J.COPSYC.2019.01.002. 30731260 PMC6625938

[bib13] Blangero, A., Khan, A., Rode, G., Rossetti, Y., & Pisella, L. (2011). Dissociation between intentional and automatic remapping: Different levels of inter-hemispheric transfer. *Vision Research,* 51(8), 932–939, 10.1016/j.visres.2011.01.012. 21316385

[bib14] Bogadhi, A. R., Bollimunta, A., Leopold, D. A., & Krauzlis, R. J. (2018). Brain regions modulated during covert visual attention in the macaque. *Scientific Reports,* 8(1), 1–15, 10.1038/s41598-018-33567-9. 30323289 PMC6189039

[bib15] Bollimunta, A., Bogadhi, A. R., & Krauzlis, R. J. (2018). Comparing frontal eye field and superior colliculus contributions to covert spatial attention. *Nature Communications,* 9(1), 1–11, 10.1038/s41467-018-06042-2. PMC6120922 30177726

[bib16] Born, S., Ansorge, U., & Kerzel, D. (2013). Predictability of spatial and non-spatial target properties improves perception in the covert interval. *Vision Research,* 91, 93–101, 10.1016/J.VISRES.2013.08.003. 23954813

[bib17] Brainard, D. H. (1997). The Psychophysics Toolbox. *Spatial Vision,* 10(4), 433–436. 9176952

[bib18] Brascamp, J. W., Pels, E., & Kristjánsson, Á. (2011). Priming of pop-out on multiple time scales during visual search. *Vision Research,* 51(17), 1972–1978, 10.1016/J.VISRES.2011.07.007. 21782839

[bib19] Brown, J. W., Hanes, D. P., Schall, J. D., & Stuphorn, V. (2008). Relation of frontal eye field activity to saccade initiation during a countermanding task. *Experimental Brain Research,* 190(2), 135–151, 10.1007/S00221-008-1455-0/FIGURES/13. 18604527 PMC2748998

[bib21] Casteau, S., & Smith, D. T. (2020). Covert attention beyond the range of eye-movements: Evidence for a dissociation between exogenous and endogenous orienting. *Cortex,* 122, 170–186, 10.1016/J.CORTEX.2018.11.007. 30528427

[bib22] Castet, E., Jeanjean, S., Montagnini, A., Laugier, D., & Masson, G. S. (2006). Dynamics of attentional deployment during saccadic programming. *Journal of Vision,* 6(3), 2–2, 10.1167/6.3.2. 16643090

[bib23] Castiello, U., & Umiltà, C. (1990). Size of the attentional focus and efficiency of processing. *Acta Psychologica,* 73(3), 195–209, 10.1016/0001-6918(90)90022-8. 2353586

[bib24] Chometton, S., Charrière, K., Bayer, L., Houdayer, C., Franchi, G., Poncet, F., & Risold, P. Y. (2017). The rostromedial zona incerta is involved in attentional processes while adjacent LHA responds to arousal: c-Fos and anatomical evidence. *Brain Structure and Function,* 222(6), 2507–2525, 10.1007/S00429-016-1353-3/FIGURES/10. 28185007

[bib25] Cromer, J. A., & Waitzman, D. M. (2006). Neurones associated with saccade metrics in the monkey central mesencephalic reticular formation. *The Journal of Physiology,* 570(3), 507–523, 10.1113/JPHYSIOL.2005.096834. 16308353 PMC1479872

[bib26] Deubel, H. (2008). The time course of presaccadic attention shifts. *Psychological Research,* 72(6), 630–640, 10.1007/S00426-008-0165-3/FIGURES/5. 18839208

[bib27] Deubel, H., & Schneider, W. X. (1996). Saccade target selection and object recognition: Evidence for a common attentional mechanism. *Vision Research,* 36(12), 1827–1837, 10.1016/0042-6989(95)00294-4. 8759451

[bib28] Dhawan, S., Deubel, H., & Jonikaitis, D. (2013). Inhibition of saccades elicits attentional suppression. *Journal of Vision,* 13(6), 9–9, 10.1167/13.6.9. 23685392

[bib29] Diamond, M. R., Ross, J., & Morrone, M. C. (2000). Extraretinal Control of Saccadic Suppression. *Journal of Neuroscience,* 20(9), 3449–3455, 10.1523/JNEUROSCI.20-09-03449.2000. 10777808 PMC6773104

[bib30] Dorris, M. C., Olivier, E., & Munoz, D. P. (2007). Competitive integration of visual and preparatory signals in the superior colliculus during saccadic programming. *Journal of Neuroscience,* 27(19), 5053–5062, 10.1523/JNEUROSCI.4212-06.2007. 17494691 PMC6672386

[bib31] Eriksen, C. W., & Hoffman, J. E. (1973). The extent of processing of noise elements during selective encoding from visual displays. *Perception & Psychophysics,* 14(1), 155–160, 10.3758/BF03198630.

[bib32] Eriksen, C. W., & Hoffman, J. E. (1974). Selective attention: Noise suppression or signal enhancement? *Bulletin of the Psychonomic Society,* 4(6), 587–589, 10.3758/BF03334301.

[bib33] Faul, F., Erdfelder, E., Lang, A. G., & Buchner, A. (2007). G*Power 3: A flexible statistical power analysis program for the social, behavioral, and biomedical sciences. *Behavior Research Methods,* 39(2), 175–191, 10.3758/BF03193146. 17695343

[bib34] Fecteau, J. H., & Munoz, D. P. (2006). Salience, relevance, and firing: a priority map for target selection. *Trends in Cognitive Sciences,* 10(8), 382–390, 10.1016/j.tics.2006.06.011. 16843702

[bib35] Filali-Sadouk, N., Castet, E., Olivier, E., & Zenon, A. (2010). Similar effect of cueing conditions on attentional and saccadic temporal dynamics. *Journal of Vision,* 10(4), 21–21, 10.1167/10.4.21. 20465340

[bib36] Findlay, J. M., & Walker, R. (1999). A model of saccade generation based on parallel processing and competitive inhibition. *Behavioural and Brain Sciences,* 22, 661–721. 10.1017/s0140525x99002150 11301526

[bib37] Fischer, B., & Ramsperger, E. (1984). Human express saccades: extremely short reaction times of goal directed eye movements. *Experimental Brain Research,* 57(1), 191–195, 10.1007/BF00231145. 6519226

[bib38] Fischer, B., & Ramsperger, E. (1986). Human express saccades: effects of randomization and daily practice. *Experimental Brain Research,* 64(3), 569–578, 10.1007/BF00340494. 3803492

[bib39] Fischer, B., & Weber, H. (1997). Effects of stimulus conditions on the performance of antisaccades in man. *Experimental Brain Research,* 116(2), 191–200, 10.1007/PL00005749. 9348120

[bib40] Friedman-Hill, S. R., Robertson, L. C., Desimone, R., & Ungerleider, L. G. (2003). Posterior parietal cortex and the filtering of distractors. *Proceedings of the National Academy of Sciences of the United States of America,* 100(7), 4263–4268, 10.1073/PNAS.0730772100/ASSET/E9B16523-999D-43B1-8F9A-9EA64B5CEE01/ASSETS/GRAPHIC/PQ0730772005.JPEG. 12646699 PMC153081

[bib41] Gaspelin, N., Leonard, C. J., & Luck, S. J. (2015). Direct Evidence for Active Suppression of Salient-but-Irrelevant Sensory Inputs. *Psychological Science,* 26(11), 1740–1750, 10.1177/0956797615597913. 26420441 PMC4922750

[bib42] Gaspelin, N., & Luck, S. J. (2018). The Role of Inhibition in Avoiding Distraction by Salient Stimuli. *Trends in Cognitive Sciences,* 22(1), 79–92, 10.1016/j.tics.2017.11.001. 29191511 PMC5742040

[bib43] Geng, J. J. (2014). Attentional Mechanisms of Distractor Suppression. *Current Directions in Psychological Science,* 23(2), 147–153, 10.1177/0963721414525780.

[bib44] Gillebert, C. R., Mantini, D., Thijs, V., Sunaert, S., Dupont, P., & Vandenberghe, R. (2011). Lesion evidence for the critical role of the intraparietal sulcus in spatial attention. *Brain,* 134(6), 1694–1709, 10.1093/BRAIN/AWR085. 21576110

[bib46] Goldberg, M. E., Bisley, J. W., Powell, K. D., & Gottlieb, J. (2006). Saccades, salience and attention: the role of the lateral intraparietal area in visual behavior. *Progress in Brain Research,* 155(B), 157–175, 10.1016/S0079-6123(06)55010-1. 17027387 PMC3615538

[bib47] Gregoriou, G. G., Gotts, S. J., & Desimone, R. (2012). Cell-Type-Specific Synchronization of Neural Activity in FEF with V4 during Attention. *Neuron,* 73(3), 581–594, 10.1016/J.NEURON.2011.12.019. 22325208 PMC3297082

[bib48] Hanning, N. M., Szinte, M., & Deubel, H. (2019). Visual attention is not limited to the oculomotor range. *Proceedings of the National Academy of Sciences of the United States of America,* 116(19), 9665–9670, 10.1073/PNAS.1813465116/SUPPL_FILE/PNAS.1813465116.SM02.MP4. 31004064 PMC6511044

[bib49] Harrison, W. J., Mattingley, J. B., & Remington, R. W. (2013a). Eye Movement Targets Are Released from Visual Crowding. *Journal of Neuroscience,* 33(7), 2927–2933, 10.1523/JNEUROSCI.4172-12.2013. 23407951 PMC6619226

[bib50] Harrison, W. J., Mattingley, J. B., & Remington, R. W. (2013b). Releasing crowding prior to a saccade requires more than “attention”: response to van Koningsbruggen and Buonocore. *Journal of Neuroscience*, 33. 24003451

[bib51] Harrison, W. J., Retell, J. D., Remington, R. W., & Mattingley, J. B. (2013). Visual Crowding at a Distance during Predictive Remapping. *Current Biology,* 23(9), 793–798, 10.1016/J.CUB.2013.03.050. 23562269

[bib52] Hikosaka, O., & Wurtz, R. H. (1983). Visual and oculomotor functions of monkey substantia nigra pars reticulata. III. Memory-contingent visual and saccade responses. *Journal of Neurophysiology,* 49(5), 1268–1284, 10.1152/JN.1983.49.5.1268. 6864250

[bib53] Hikosaka, O., & Wurtz, R. H. (1985). Modification of saccadic eye movements by GABA-related substances. II. Effects of muscimol in monkey substantia nigra pars reticulata. *Journal of Neurophysiology,* 53(1), 292–308, 10.1152/JN.1985.53.1.292. 2983038

[bib54] Hoffman, J. E. (1975). Hierarchical stages in the processing of visual information. *Perception & Psychophysics,* 18(5), 348–354, 10.3758/BF03211211.

[bib55] Hoffman, J. E., & Nelson, B. (1981). Spatial selectivity in visual search. *Perception & Psychophysics,* 30(3), 283–290, 10.3758/BF03214284. 7322804

[bib56] Hoffman, J. E., & Subramaniam, B. (1995a). The role of visual attention in saccadic eye movements. *Perception & Psychophysics,* 57(6), 787–795, 10.3758/BF03206794. 7651803

[bib57] Hoffman, J. E., & Subramaniam, B. (1995b). The role of visual attention in saccadic eye movements. *Perception & Psychophysics,* 57(6), 787–795, 10.3758/BF03206794. 7651803

[bib58] Ignashchenkova, A., Dicke, P. W., Haarmeier, T., & Thier, P. (2003). Neuron-specific contribution of the superior colliculus to pre-saccadic and covert shifts of attention. *Nature Neuroscience,* 7(1), 56–64, 10.1038/nn1169. 14699418

[bib59] Itti, L., & Koch, C. (2000). A saliency-based search mechanism for pre-saccadic and covert shifts of visual attention. *Vision Research,* 40(10–12), 1489–1506, 10.1016/S0042-6989(99)00163-7. 10788654

[bib60] Itti, L., & Koch, C. (2001). Computational modelling of visual attention. *Nature Reviews Neuroscience,* 2(3), 194–203, 10.1038/35058500. 11256080

[bib61] Juan, C. H., Shorter-Jacobi, S. M., & Schall, J. D. (2004). Dissociation of spatial attention and saccade preparation. *Proceedings of the National Academy of Sciences of the United States of America,* 101(43), 15541–15544, 10.1073/pnas.0403507101. 15489272 PMC524443

[bib62] Kastner, S., de Weerd, P., Desimone, R., & Ungerleider, L. G. (1998). Mechanisms of directed attention in the human extrastriate cortex as revealed by functional MRI. *Science,* 282(5386), 108–111, 10.1126/science.282.5386.108. 9756472

[bib63] Kastner, S., & Pinsk, M. A. (2004). Visual attention as a multilevel selection process. *Cognitive, Affective, & Behavioral Neuroscience,* 4(4), 483–500, 10.3758/CABN.4.4.483. 15849892

[bib64] Keller, E. L. (1974). Participation of medial pontine reticular formation in eye movement generation in monkey. *Journal of Neurophysiology,* 37(2), 316–332, 10.1152/JN.1974.37.2.316. 4205567

[bib65] Keller, E. L. (1977). Control of saccadic eye movements by midline brain stem neurons. In R., Baker, & A. Berthoz (Eds.), *Control of Gaze by Brain Stem Neurons* (pp. 327–336). St. Louis: Elsevier.

[bib66] Khan, A. Z., Blangero, A., Rossetti, Y., Salemme, R., Luauté, J., Deubel, H., & Pisella, L. (2009). Parietal Damage Dissociates Saccade Planning from Presaccadic Perceptual Facilitation. *Cerebral Cortex,* 19(2), 383–387, 10.1093/CERCOR/BHN088. 18534990

[bib67] Khan, A. Z., Blohm, G., McPeek, R. M., & Lefèvre, P. (2009). Differential influence of attention on gaze and head movements. *Journal of Neurophysiology,* 101(1), 198–206, 10.1152/JN.90815.2008/ASSET/IMAGES/LARGE/Z9K0010992440005.JPEG. 18987122 PMC2637015

[bib68] Khan, A. Z., Blohm, G., Pisella, L., & Munoz, D. P. (2015). Saccade execution suppresses discrimination at distractor locations rather than enhancing the saccade goal location. *European Journal of Neuroscience,* 41(12), 1624–1634, 10.1111/ejn.12923. 25891002

[bib69] Khan, A. Z., Munoz, D. P., Takahashi, N., Blohm, G., & McPeek, R. M. (2016). Effects of a pretarget distractor on saccade reaction times across space and time in monkeys and humans. *Journal of Vision,* 16(7), 1–20, 10.1167/16.7.5. PMC5833323 27148697

[bib70] Knöll, J., Binda, P., Concetta Morrone, M., & Bremmer, F. (2011). Spatiotemporal profile of peri-saccadic contrast sensitivity. *Journal of Vision,* 11(14), 15–15, 10.1167/11.14.15. 22178703

[bib71] Kowler, E., Anderson, E., Dosher, B., & Blaser, E. (1995). The role of attention in the programming of saccades. *Vision Research,* 35(13), 1897–1916, 10.1016/0042-6989(94)00279-U. 7660596

[bib73] Krauzlis, R. J., Liston, D., & Carello, C. D. (2004). Target selection and the superior colliculus: goals, choices and hypotheses. *Vision Research,* 44(12), 1445–1451, 10.1016/J.VISRES.2004.01.005. 15066403

[bib74] Krauzlis, R. J., Lovejoy, L. P., & Zénon, A. (2013). Superior Colliculus and Visual Spatial Attention. *Annual Review of Neuroscience,* 36, 165–182, 10.1146/ANNUREV-NEURO-062012-170249. PMC3820016 23682659

[bib75] Kristjánsson, Á., & Ásgeirsson, Á. G. (2019). Attentional priming: recent insights and current controversies. *Current Opinion in Psychology,* 29, 71–75, 10.1016/J.COPSYC.2018.11.013. 30553136

[bib77] Laberge, D., & Brown, V. (1986). Variations in size of the visual field in which targets are presented: An attentional range effect. *Perception & Psychophysics,* 40(3), 188–200, 10.3758/BF03203016. 3774503

[bib78] Lai, D., Brandt, S., Luksch, H., & Wessel, R. (2011). Recurrent antitopographic inhibition mediates competitive stimulus selection in an attention network. *Journal of Neurophysiology,* 105(2), 793–805, 10.1152/JN.00673.2010/ASSET/IMAGES/LARGE/Z9K0021105920010.JPEG. 21160008 PMC3059176

[bib79] Latour, P. L. (1962). Visual threshold during eye movements. *Vision Research,* 2(3), 261–262.

[bib80] Li, H. H., Barbot, A., & Carrasco, M. (2016). Saccade Preparation Reshapes Sensory Tuning. *Current Biology,* 26(12), 1564–1570, 10.1016/J.CUB.2016.04.028. 27265397 PMC4916013

[bib81] Li, H. H., Hanning, N. M., & Carrasco, M. (2021). To look or not to look: dissociating presaccadic and covert spatial attention. *Trends in Neurosciences,* 44(8), 669–686, 10.1016/J.TINS.2021.05.002. 34099240 PMC8552810

[bib82] Li, H. H., Pan, J., & Carrasco, M. (2021). Different computations underlie pre-saccadic presaccadic and covert spatial attention. *Nature Human Behaviour,* 5(10), 1418–1431, 10.1038/s41562-021-01099-4. PMC8552811 33875838

[bib83] Luschei, E. S., & Fuchs, A. F. (1972). Activity of brain stem neurons during eye movements of alert monkeys. *Journal of Neurophysiology,* 35(4), 445–461, 10.1152/JN.1972.35.4.445. 4624736

[bib84] Mackeben, M. (1999). Sustained focal attention and peripheral letter recognition. *Spatial Vision,* 12(1), 51–72, 10.1163/156856899X00030. 10195388

[bib85] Marino, R. A., Trappenberg, T. P., Dorris, M., & Munoz, D. P. (2012). Spatial Interactions in the Superior Colliculus Predict Saccade Behavior in a Neural Field Model. *Journal of Cognitive Neuroscience,* 24(2), 315–336, 10.1162/JOCN_A_00139. 21942761

[bib86] Mayfrank, L., Mobashery, M., Kimmig, H., & Fischer, B. (1986). The role of fixation and visual attention in the occurrence of express saccades in man. *European Archives of Psychiatry and Neurological Sciences,* 235(5), 269–275, 10.1007/BF00515913. 3732337

[bib87] McPeek, R. M. (2006). Incomplete suppression of distractor-related activity in the frontal eye field results in curved saccades. *Journal of Neurophysiology,* 96(5), 2699–2711, 10.1152/JN.00564.2006/ASSET/IMAGES/LARGE/Z9K0110677500011.JPEG. 16885521 PMC1876735

[bib88] McPeek, R. M., & Keller, E. L. (2002). Superior colliculus activity related to concurrent processing of saccade goals in a visual search task. *Journal of Neurophysiology,* 87(4), 1805–1815, 10.1152/JN.00501.2001/ASSET/IMAGES/LARGE/9K0422250007.JPEG. 11929902

[bib89] McPeek, R. M., & Keller, E. L. (2004). Deficits in saccade target selection after inactivation of superior colliculus. *Nature Neuroscience,* 7(7), 757–763, 10.1038/nn1269. 15195099

[bib90] McSorley, E., Haggard, P., & Walker, R. (2004). Distractor modulation of saccade trajectories: Spatial separation and symmetry affects. *Experimental Brain Research,* 155(3), 320–333, 10.1007/S00221-003-1729-5/FIGURES/7. 14726987

[bib91] McSorley, E., Haggard, P., & Walker, R. (2006). Time course of oculomotor inhibition revealed by saccade trajectory modulation. *Journal of Neurophysiology,* 96(3), 1420–1424, 10.1152/jn.00315.2006. 16624996

[bib92] Mikula, L., Jacob, M., Tran, T., Pisella, L., & Khan, A. Z. (2018). Spatial and temporal dynamics of presaccadic attentional facilitation before pro- and antisaccades. *Journal of Vision,* 18(11), 1–16, 10.1167/18.11.2. 30326049

[bib93] Mirpour, K., Arcizet, F., Ong, W. S., & Bisley, J. W. (2009). Been there, seen that: A neural mechanism for performing efficient visual search. *Journal of Neurophysiology,* 102(6), 3481–3491, 10.1152/jn.00688.2009. 19812286 PMC2804407

[bib94] Mitrofanis, J. (2005). Some certainty for the “zone of uncertainty”? Exploring the function of the zona incerta. *Neuroscience,* 130(1), 1–15, 10.1016/J.NEUROSCIENCE.2004.08.017. 15561420

[bib95] Moore, T., & Armstrong, K. M. (2003). Selective gating of visual signals by microstimulation of frontal cortex. *Nature,* 421(6921), 370–373, 10.1038/nature01341. 12540901

[bib96] Moore, T., & Fallah, M. (2001). Control of eye movements and spatial attention. *Proceedings of the National Academy of Sciences of the United States of America,* 98(3), 1273–1276, 10.1073/PNAS.98.3.1273. 11158629 PMC14744

[bib97] Moore, T., & Fallah, M. (2004). Microstimulation of the frontal eye field and its effects on covert spatial attention. *Journal of Neurophysiology,* 91(1), 152–162, 10.1152/JN.00741.2002. 13679398

[bib98] Müller, M. M., Teder-Sälejärvi, W., & Hillyard, S. A. (1998). The time course of cortical facilitation during cued shifts of spatial attention. *Nature Neuroscience,* 1(7), 631–634, 10.1038/2865. 10196572

[bib99] Munoz, D. P., & Fecteau, J. H. (2002). Vying for dominance: dynamic interactions control visual fixation and saccadic initiation in the superior colliculus. *Progress in Brain Research,* 140, 3–19, 10.1016/S0079-6123(02)40039-8. 12508579

[bib100] Murthy, A., Thompson, K. G., & Schall, J. D. (2001). Dynamic dissociation of visual selection from saccade programming in frontal eye field. *Journal of Neurophysiology,* 86(5), 2634–2637, 10.1152/JN.2001.86.5.2634. 11698551

[bib101] Mysore, S. P., Asadollahi, A., & Knudsen, E. I. (2010). Global Inhibition and Stimulus Competition in the Owl Optic Tectum. *Journal of Neuroscience,* 30(5), 1727–1738, 10.1523/JNEUROSCI.3740-09.2010. 20130182 PMC2828882

[bib102] Mysore, S. P., & Knudsen, E. I. (2011). The role of a midbrain network in competitive stimulus selection. *Current Opinion in Neurobiology,* 21(4), 653–660, 10.1016/J.CONB.2011.05.024. 21696945 PMC3177965

[bib103] Mysore, S. P., & Knudsen, E. I. (2013). A shared inhibitory circuit for both exogenous and endogenous control of stimulus selection. *Nature Neuroscience,* 16(4), 473–478, 10.1038/nn.3352. 23475112 PMC3609877

[bib104] Nummela, S. U., & Krauzlis, R. J. (2010). Inactivation of primate superior colliculus biases target choice for smooth pursuit, saccades, and button press responses. *Journal of Neurophysiology,* 104(3), 1538–1548, 10.1152/JN.00406.2010/ASSET/IMAGES/LARGE/Z9K0091003420009.JPEG. 20660420 PMC2944695

[bib105] Ouerfelli-Ethier, J., Salemme, R., Fournet, R., Urquizar, C., Pisella, L., Khan, A. Z., et al (2021). Impaired Spatial Inhibition Processes for Interhemispheric Anti-saccades following Dorsal Posterior Parietal Lesions. *Cerebral Cortex Communications,* 2, 1–15, 10.1093/texcom/tgab054. PMC8481671 34604753

[bib106] Paltoglou, A. E., & Neri, P. (2012). Attentional control of sensory tuning in human visual perception. *Journal of Neurophysiology,* 107(5), 1260–1274, 10.1152/JN.00776.2011/ASSET/IMAGES/LARGE/Z9K0031211980009.JPEG. 22131380 PMC3311683

[bib107] Pestilli, F., & Carrasco, M. (2005). Attention enhances contrast sensitivity at cued and impairs it at uncued locations. *Vision Research,* 45(14), 1867–1875, 10.1016/J.VISRES.2005.01.019. 15797776

[bib108] Posner, M. I. (1980). Orienting of Attention. *Quarterly Journal of Experimental Psychology,* 32(1), 3–25, 10.1080/00335558008248231. 7367577

[bib109] Posner, M. I., & Cohen, Y. (1984). Components of visual orienting. In H., Bouma & D. G., Bouwhuis (Eds.), *Attention and performance X* (Vol. 10, pp. 531–556). Hillsdale, NJ: Lawrence Erlbaum Associates.

[bib110] Posner, M. I., Nissen, M. J., & Ogden, W. C. (1978). Attended and unattended processing modes: The role of set for spatial location. In H. L., Pick & I. J., Saltzman (Eds.), *Modes of perceiving and processing information* (pp. 137–158). Hillsdale, NJ: Lawrence Erlbaum Associates.

[bib111] Ptak, R. (2012). The frontoparietal attention network of the human brain: action, saliency, and a priority map of the environment. *The Neuroscientist : A Review Journal Bringing Neurobiology, Neurology and Psychiatry,* 18(5), 502–515, 10.1177/1073858411409051. 21636849

[bib112] Ray, S., Pouget, P., & Schall, J. D. (2009). Functional distinction between visuomovement and movement neurons in macaque frontal eye field during saccade countermanding. *Journal of Neurophysiology,* 102(6), 3091–3100, 10.1152/JN.00270.2009/ASSET/IMAGES/LARGE/Z9K0120997980004.JPEG. 19776364 PMC2804409

[bib113] Reynolds, J. H., & Chelazzi, L. (2004). Attentional Modulation of Visual Processing. *Annual Review of Neuroscience,* 27, 611–658, 10.1146/annurev.neuro.26.041002.131039. 15217345

[bib114] Reynolds, J. H., Chelazzi, L., & Desimone, R. (1999). Competitive mechanisms subserve attention in macaque areas V2 and V4. *Journal of Neuroscience,* 19(5), 1736–1753, 10.1523/jneurosci.19-05-01736.1999. 10024360 PMC6782185

[bib115] Rizzolatti, G., & Craighero, L. (1998). Spatial attention: Mechanisms and theories. In M. Sabourin, F. Craik, & M. Robert (Eds.), *Advances in psychological science* (Vol. 2, pp. 171–198). London: Psychology Press.

[bib116] Rizzolatti, G., Riggio, L., Dascola, I., & Umiltá, C. (1987). Reorienting attention across the horizontal and vertical meridians: Evidence in favor of a premotor theory of attention. *Neuropsychologia,* 25(1), 31–40, 10.1016/0028-3932(87)90041-8. 3574648

[bib117] Ro, T., Farnè, A., & Chang, E. (2003). Inhibition of return and the human frontal eye fields. *Experimental Brain Research,* 150, 290–296, 10.1007/s00221-003-1470-0. 12692701

[bib118] Rolfs, M., & Carrasco, M. (2012). Rapid Simultaneous Enhancement of Visual Sensitivity and Perceived Contrast during Saccade Preparation. *Journal of Neuroscience,* 32(40), 13744–13752a, 10.1523/JNEUROSCI.2676-12.2012. 23035086 PMC3498617

[bib119] Sakai, K., Rowe, J. B., & Passingham, R. E. (2002). Active maintenance in prefrontal area 46 creates distractor-resistant memory. *Nature Neuroscience,* 5(5), 479–484, 10.1038/nn846. 11953754

[bib121] Schall, J. D. (2002). The neural selection and control of saccades by the frontal eye field. *Philosophical Transactions of the Royal Society of London. Series B: Biological Sciences,* 357(1424), 1073–1082, 10.1098/RSTB.2002.1098. 12217175 PMC1693021

[bib122] Schall, J. D., Hanes, D. P., Thompson, K. G., & King, D. J. (1995). Saccade target selection in frontal eye field of macaque. I. Visual and premovement activation. *Journal of Neuroscience,* 15(10), 6905–6918, 10.1523/jneurosci.15-10-06905.1995. 7472447 PMC6577995

[bib123] Schlag, J., Dassonville, P., & Schlag-Rey, M. (1998). Interaction of the two frontal eye fields before saccade onset. *Journal of Neurophysiology,* 79(1), 64–72, 10.1152/JN.1998.79.1.64/ASSET/IMAGES/LARGE/JNP.JA35F8.JPEG. 9425177

[bib124] Schlag-Rey, M., Schlag, J., & Dassonville, P. (1992). How the frontal eye field can impose a saccade goal on superior colliculus neurons. *Journal of Neurophysiology,* 67(4), 1003–1005, 10.1152/JN.1992.67.4.1003. 1588383

[bib125] Serences, J. T., Yantis, S., Culberson, A., & Awh, E. (2004). Preparatory activity in visual cortex indexes distractor suppression during covert spatial orienting. *Journal of Neurophysiology,* 92(6), 3538–3545, 10.1152/jn.00435.2004. 15254075

[bib127] Śmigasiewicz, K., Weinrich, J., Reinhardt, B., & Verleger, R. (2014). Deployment and release of interhemispheric inhibition in dual-stream rapid serial visual presentation. *Biological Psychology,* 99(1), 47–59, 10.1016/J.BIOPSYCHO.2014.02.008. 24576590

[bib128] Smith, D. T., Jackson, S. R., & Rorden, C. (2005). Transcranial magnetic stimulation of the left human frontal eye fields eliminates the cost of invalid endogenous cues. *Neuropsychologia,* 43(9), 1288–1296, 10.1016/J.NEUROPSYCHOLOGIA.2004.12.003. 15949513

[bib129] Smith, D. T., Schenk, T., & Rorden, C. (2012). Saccade preparation is required for exogenous attention but not endogenous attention or IOR. *Journal of Experimental Psychology: Human Perception and Performance,* 38(6), 1447, 10.1037/A0027794. 22428677

[bib130] Sommer, M. A., & Wurtz, R. H. (2000). Composition and topographic organization of signals sent from the frontal eye field to the superior colliculus. *Journal of Neurophysiology,* 83(4), 1979–2001, 10.1152/JN.2000.83.4.1979/ASSET/IMAGES/LARGE/9K0400907014.JPEG. 10758109

[bib131] Striemer, C., Blangero, A., Rossetti, Y., Boisson, D., Rode, G., Vighetto, A., & Danckert, J. (2007). Deficits in peripheral visual attention in patients with optic ataxia. *NeuroReport,* 18(11), 1171–1175, 10.1097/WNR.0b013e32820049bd. 17589321

[bib132] Striemer, C., Locklin, J., Blangero, A., Rossetti, Y., Pisella, L., & Danckert, J. (2009). Attention for action?. Examining the link between attention and visuomotor control deficits in a patient with optic ataxia. *Neuropsychologia,* 47(6), 1491–1499, 10.1016/j.neuropsychologia.2008.12.021. 19154751

[bib133] Suzuki, M., & Gottlieb, J. (2013). Distinct neural mechanisms of distractor suppression in the frontal and parietal lobe. *Nature Neuroscience,* 16(1), 98–104, 10.1038/nn.3282. 23242309 PMC4207121

[bib134] Sylvester, C. M., Jack, A. I., Corbetta, M., & Shulman, G. L. (2008). Anticipatory Suppression of Nonattended Locations in Visual Cortex Marks Target Location and Predicts Perception. *Journal of Neuroscience,* 28(26), 6549–6556, 10.1523/JNEUROSCI.0275-08.2008. 18579728 PMC2587329

[bib135] Szczepanski, S. M., & Kastner, S. (2013). Shifting Attentional Priorities: Control of Spatial Attention through Hemispheric Competition. *Journal of Neuroscience,* 33(12), 5411–5421, 10.1523/JNEUROSCI.4089-12.2013. 23516306 PMC3651512

[bib136] Szczepanski, S. M., Konen, C. S., & Kastner, S. (2010). Mechanisms of Spatial Attention Control in Frontal and Parietal Cortex. *Journal of Neuroscience,* 30(1), 148–160, 10.1523/JNEUROSCI.3862-09.2010. 20053897 PMC2809378

[bib137] Thompson, K. G., & Bichot, N. P. (2005). A visual salience map in the primate frontal eye field. *Progress in Brain Research,* 147(SPEC. ISS.), 249–262, 10.1016/S0079-6123(04)47019-8. 15581711

[bib138] Thompson, K. G., Bichot, N. P., & Schall, J. D. (1997). Dissociation of Visual Discrimination From Saccade Programming in Macaque Frontal Eye Field. *Journal of Neurophysiology,* 77(2), 1046–1050, 10.1152/jn.1997.77.2.1046. 9065870

[bib139] Thompson, K. G., Biscoe, K. L., & Sato, T. R. (2005). Neuronal Basis of Covert Spatial Attention in the Frontal Eye Field. *Journal of Neuroscience,* 25(41), 9479–9487, 10.1523/JNEUROSCI.0741-05.2005. 16221858 PMC2804969

[bib142] Volkmann, F. C., Riggs, L. A., White, K. D., & Moore, R. K. (1978). Contrast sensitivity during saccadic eye movements. *Vision Research,* 18(9), 1193–1199, 10.1016/0042-6989(78)90104-9. 716239

[bib143] Walker, R., Techawachirakul, P., & Haggard, P. (2009). Frontal eye field stimulation modulates the balance of salience between target and distractors. *Brain Research,* 1270, 54–63, 10.1016/J.BRAINRES.2009.02.081. 19285965

[bib144] Wang, L., Herman, J. P., & Krauzlis, R. J. (2022). Neuronal modulation in the mouse superior colliculus during covert visual selective attention. *Scientific Reports,* 12(1), 1–16, 10.1038/s41598-022-06410-5. 35169189 PMC8847498

[bib145] Wang, Y., Major, D. E., & Karten, H. J. (2004). Morphology and connections of nucleus isthmi pars magnocellularis in chicks (Gallus gallus). *Journal of Comparative Neurology,* 469(2), 275–297, 10.1002/CNE.11007. 14694539

[bib146] Wardak, C., Ibos, G., Duhamel, J. R., & Olivier, E. (2006). Contribution of the Monkey Frontal Eye Field to Covert Visual Attention. *Journal of Neuroscience,* 26(16), 4228–4235, 10.1523/JNEUROSCI.3336-05.2006. 16624943 PMC6674003

[bib147] Wardak, C., Olivier, E., & Duhamel, J. R. (2011). The relationship between spatial attention and saccades in the frontoparietal network of the monkey. *European Journal of Neuroscience,* 33(11), 1973–1981, 10.1111/J.1460-9568.2011.07710.X. 21645093

[bib148] Weber, H., Aiple, F., Fischer, B., & Latanov, A. (1992). Experimental Brain Research Dead zone for express saeeades. *Exp Brain Res,* 89, 214–222. 1601099 10.1007/BF00229018

[bib149] Wurtz, R. H., & Mohler, C. W. (1976). Enhancement of visual responses in monkey striate cortex and frontal eye fields. *Journal of Neurophysiology,* 39(4), 766–772, 10.1152/JN.1976.39.4.766. 823304

[bib152] Zuber, B. L., & Stark, L. (1966). Saccadic suppression: Elevation of visual threshold associated with saccadic eye movements. *Experimental Neurology,* 16(1), 65–79, 10.1016/0014-4886(66)90087-2. 5923485

